# Investigation of genetic polymorphism of Russian rape and turnip rape varieties using SSR and SRAP markers

**DOI:** 10.18699/VJGB-22-42

**Published:** 2022-07

**Authors:** I.A. Klimenko, V.T. Volovik, A.A. Antonov, V.A. Dushkin, A.O. Shamustakimova, Yu.M. Yu.M. Mavlyutov

**Affiliations:** Federal Williams Research Center of Forage Production and Agroecology, Lobnya, Moscow region, Russia; Federal Williams Research Center of Forage Production and Agroecology, Lobnya, Moscow region, Russia; Federal Williams Research Center of Forage Production and Agroecology, Lobnya, Moscow region, Russia; Federal Williams Research Center of Forage Production and Agroecology, Lobnya, Moscow region, Russia; Federal Williams Research Center of Forage Production and Agroecology, Lobnya, Moscow region, Russia; Federal Williams Research Center of Forage Production and Agroecology, Lobnya, Moscow region, Russia

**Keywords:** forage crops, Brassica napus L., B. rapa L. campestris, bulk samples, genetic polymorphism, SSR markers, SRAP markers, кормовые культуры, Brassica napus L., B. rapa L. campestris, «балк-образцы», генетический полиморфизм, SSR-маркеры, SRAP-маркеры

## Abstract

Rapeseed (Brassica napus L.) and turnip rape (B. rapa L. subsp. campestris (L.)) are important agricultural plants widely used for food, fodder and technical purposes and as green manure. Over the past decades, a large number of perspective varieties that are being currently cultivated in every region of Russia have been developed. To increase the breeding eff iciency and facilitate the seed production, modern molecular-genetic techniques should be introduced as means to estimate species and varietal diversity. The objective of the presented research study was to investigate DNA polymorphism of the rapeseed and turnip rape varieties developed at Federal Williams Research Center of Forage Production and Agroecology and detect informative markers for varietal identif ication and genetic certif ication. To genotype 18 gDNA samples, 42 and 25 combinations of respective SSR and SRAP primers were used. The results obtained demonstrate that SRAP markers were more effective for polymorphism analysis: 36 % of the tested markers revealed genetic polymorphism compared with only 16.7 % of microsatellite loci. Molecular markers to detect differences at interspecif ic and intervarietal levels have also been found. For the investigated set, such microsatellite loci as Na12A02, Ni2C12, Ni02-D08a, Ra02-E01, Ni03H07а and SRAP-marker combinations as F13-R9, Me4- R7, F11-Em2, F10-R7, F9-Em2 and F9-R8 proved to be informative. Application of the two marker techniques made it possible to detect a higher level of DNA polymorphism in plants of different types (spring and winter varieties) if compared against the intervarietal differences within a species or a group. According to Nei’s genetic diversity index, in the cluster of winter rapeseed, VIK 2 and Gorizont varieties had the longest genetic distance, and in the spring cluster, these were Novosel and Veles. A high level of similarity was found between Vikros and Bizon winter rapeseed varieties. The results obtained have a high practical value for varietal specif ication of seed material and genetic certif ication of rapeseed and turnip rape varieties.

## Abstract (ru)

Рапс (Brassica napus L.) и сурепица (B. rapa L. subsp. campestris (L.)) – важные сельскохозяйственные культуры, широко используются для продовольственных, кормовых и технических целей, а также в качестве сидератов. За последние десятилетия создано большое количество перспективных сортов, культивируемых практически во всех регионах России. Для повышения эффективности селекционного процесса и успешного развития семеноводства необходимо внедрять современные молекулярно-генетические методы оценки видового и сортового разнообразия. Цель настоящей работы заключалась в изучении ДНК-полиморфизма сортов рапса и сурепицы селекции Федерального научного центра кормопроизводства и агроэкологии им. В.Р. Вильямса и выявлении информативных маркеров для сортовой идентификации и генетической паспортизации. Для генотипирования 18 образцов геномной ДНК использовали 42 и 25 комбинаций SSR- и SRAP-праймеров соответственно. Результаты показали, что маркеры SRAP более эффективны для анализа полиморфизма изучаемого материала: 36 % от общего числа испытанных маркеров демонстрировали генетический полиморфизм, тогда как для микросателлитных локусов этот показатель равнялся 16.7 %. Определены молекулярные маркеры для выявления различий на межвидовом и межсортовом уровнях. Информативными для исследуемой выборки сортов оказались микросателлитные локусы Na12A02, Ni2C12, Ni02-D08a, Ra02-E01, Ni03H07а и комбинации SRAP-маркеров F13-R9, Me4-R7, F11-Em2, F10-R7, F9-Em2 и F9-R8. Анализ сортового материала по двум системам маркирования показал более высокий уровень ДНК-полиморфизма у образцов растений разного типа развития (яровой/озимый) в сравнении с различиями между сортами в пределах вида или группы. Согласно индексам генетического разнообразия Нея, в кластере сортов озимого рапса наибольшей генетической удаленностью выделялись ВИК 2 и Горизонт, среди яровых – Новосёл и Велес. Высокий уровень сходства обнаружен между яровыми сортами рапса Викрос и Бизон. Полученная информация имеет практическое значение для контроля сортовой принадлежности и генетической паспортизации семенного материала сортов рапса и сурепицы.

## Introduction

Cabbage oilseed crops such as rapeseed (Brassica napus L.) and turnip rape (B. rapa L. subsp. campestris (L.)) are cultivated in almost every region of Russia, and, for the foreseeable future, are regarded as the main reserve for increasing the production of vegetable oil and fodder protein. These plants are widely used in food, fodder, technical purposes and as green manure that increases soil fertility thanks to the plants’ root remains containing up to 6 tons of organic maters, 80 kg of nitrogen, 60 kg of phosphorus and 90 kg of potassium per hectare. As for their food and fodder properties, rapeseed and turnip rape exceed many other cultivated crops since their seeds are 40–48 % fat and 21–33 % protein and contain a high amount of essential amino acids (Volovik, 2015). Rapeseed can provide livestock with green forage from early spring to late fall thanks to their cold hardiness and fast regrowth after mowing. They are also an excellent silage material, and their seeds and seed by-pass products are processed to produce seed cake and coarse meal. In the recent years the varieties of rapeseed and turnip rape with low or no erucic-acid content became available and seed production has increased more than 7 times to reach the world’s third place after soybeans and cotton. Russia’s short-term plans are to increase rapeseed planting acreage to 2.5 mln he.

As for Russian research institutions working intensely to select cabbage oilseed crops, the leading ones are All-Russian Research Institute of Rapeseed, All-Russian Research Institute of Oilseed Crops and All-Russian Williams Fodder Research Institute. For the two last decades, they have produced the perspective varieties of rapeseed, turnip rape, white mustard and oil radish that have been recommended for oil production, livestock and poultry green forage, combination fodder, seed cake and coarse meal production. In 2021, “State Register” of the Russian Federation included 13 varieties of rapeseed and 3 varieties of turnip rape selected by Federal Williams Research Center of Forage Production and Agroecology (Kosolapov et al., 2019; State Register…, 2021).

For preservation and rational use of newly available varieties, intensification of the selection process and protection of intellectual property, modern and effective methods to estimate species and varietal diversity at a genetic level are to be introduced. One of such techniques that has been successfully applied in the recent years is molecular DNA markers, which, if compared against the traditional morphological indicators, possess a number of advantages. These include a high level of polymorphism; even genome distribution; reliability; a possibility to automate the assay procedure that does not depend on environmental conditions or a plant development phase (Agarwal et al., 2008; Khlestkina, 2011; Chesnokov, 2018). If the most informative and convenient DNA markers are selected, their capabilities to estimate the genetic variability of selection material are regarded as unlimited.

Laboratory for Molecular and Genetic Studies in Federal Williams Research Center of Forage Production and Agroecology has been developing a system for DNA identification and genetic certification of Russian fodder crops. For the time being, the varietal identification techniques have been adapted for perennial legume grasses such as red clover and different species of alfalfa (Klimenko et al., 2020a, b). The assay uses samples of the summary total DNA obtained through a modified method from an arbitrary selected sample of every variety’s germinants. Two types of molecular markers were used: SSR (simple sequence repeats), which detect the variability of microsatellite genome sequences, and SRAP (sequence related amplified polymorphism), which is based on PCR with a pair of primers for amplification of intron/exon regions (open reading frames). The techniques have been tested on different species of fodder crops to optimize the amplification conditions, detection and analysis of results.

A problem of reliable varietal identification is particularly topical for rapeseed due to its limited genetic variability conditioned by the intensive selection aimed at higher content and quality of oil. Currently, a significant number of published studies have been devoted to using different DNA markers for estimation of the genetic diversity of rapeseed varieties and hybrids (Plieske, Struss, 2001; Snowdon, Friedt, 2004; Klyachenko et al., 2018; Mozgova et al., 2019); to genetic mapping (Piquemal et al., 2005; Gao et al., 2007; Geng, 2012) and marking the genes of economically valuable traits (Chen et al., 2010; Ananga et al., 2012). However, only a few such studies have investigated Russian varieties. Four varieties of winter and spring rapeseed (Podmoskovniy, Vikros, VIK 2 and Severyanin) were studied by Byelorussian researchers to identify the gene alleles determining the concentration of oleic and linolic acids in rapeseed oil (Lemesh et al., 2015). The same varieties were investigated to detect the DNA markers of the genes responsible for erucic-acid synthesis (Amosova et al., 2014). Microsatellite markers were used to study the genetic polymorphism of Russian varieties Ratnik and SNK- 198 (Satina, 2010) as well as the genetic homogeneity of spring rapeseed varieties Bulat and Forward (Rogozhina et al., 2015). Such winter varieties as Stolychniy, Laureat, Gorizont, Nord and Severyanin were investigated to detect the quantitative trait loci (QTLs) associated with high winter hardiness (Mozgova et al., 2019).

The objective of the presented study was to investigate DNA polymorphism of rapeseed and turnip rape varieties developed by breeders of Federal Williams Research Center of Forage Production and Agroecology and to identify the informative markers for varietal differentiation and genetic certification.

## Materials and methods

Plant material. The study investigated 15 varieties of winter (Severyanin, Stolychniy, VIK 2, Nord, Laureat, Gorizont, Garant) and spring (Vikros, Novik, Novosel, Veles, Grant, Podmoskovniy, Lugovskoy, Bizon) rapeseed and 3 varieties of winter (Zarya) and spring (Nadezhda, Svetlana) turnip rape.

DNA extraction and PCR analysis. The gDNA was extracted from 30 germinants of each abovementioned variety (bulk samples) using the basic SDS method (Kirby, Cook, 1967; Dellaporta et al., 1983) with some modifications (Klimenko et al., 2020b). The quality and concentration of the obtained DNA fractions were verified with agarose gel (1.5 %) electrophoresis and using a Nabi spectrophotometer (MicroDigital, South Korea).

To carry out SSR analysis, 42 markers from the database Brassica info (https://www.brassica.info) and available publications were applied. The efficiency of the primers devised for these markers had been demonstrated in the studies devoted to development of the technology of rapeseed genotyping (Satina, 2010) and selection of the samples with low erucic-acid and glucosinolate content (Hasan et al., 2008). A part of the markers included in the analysis was used for hybridization control and detection of Alternaria blight resistant genotypes in Indian mustard (B. juncea L.) (Chandra et al., 2013; Sharma et al., 2018).

The PCR-mixture of 20 μl contained 3 μl 10 × PCR buffer (Taq Turbo Buffer), 0.5 μl 50 × dNTPs mix, 0.4 μl Taq polymerase (5U), forward and reverse primers (0.1 μl each, 100 μm) and 0.1 μl of DNA sample (20 ng/μl). The amplification was performed in a T-1000 thermal cycler (Bio-Rad, USA) at two different temperature regimes. The first amplification program was an initial 3-min denaturation at 95 °C followed by 30 cycles of 30 s at 94 °C, 30 s at 55–57 °C, 30 s at 72 °C and a final 5-min elongation at 72 °C (Satina, 2010). The second program included an initial 5-min denaturation at 95 °C followed by 39 cycles of 1 min at 94 °C, 2 min at 46–51 °C (depending on the primer pair in use), 2 min at 72 °C and a final 10-min elongation at 72 °C (Chandra et al., 2013). The reproducibility of obtained results was attested in three-fold replication.

SRAP analysis was carried out using 25 primer combinations comprised from 10 single oligonucleotides: F9, F13, Me4, F10, F11, R9, R7, Em2, R14, R8 (Li, Quiros, 2001; Rhouma et al., 2017). The amplification program was an initial 4-min denaturation at 94 °C followed by 10 cycles with changing temperature and duration parameters (1 min at 94 °C, 1 min at 35 °C, 1 min at 72 °C); followed by 30 cycles (1 min at 94 °C, 1 min at 50 °C, 1 min at 72 °C) and a final 5-min elongation step run at 72 °C. The PCR-mixture composition was similar to that used for the microsatellite analysis.

PCR-products were separated using 90-min 50-V agarosegel electrophoresis (4 % MetaPhorR Agarose, Rockland or 1.6 % LE, Lonza, USA). As the reference markers, 20 bp DNA Ruler (Bio-Rad), 100 kb DNA Ladder (Thermo Fisher Scientific, USA) and 100 bp + 1.5 kb (SibEnzyme, Russia) were applied.

Analysis of the obtained results. PCR-product detection and size measurement was performed using a GelDoc XR+ imaging system (Bio-Rad) and the ImageLab software (Bio- Rad Lab., Inc.) for molecular-mass markers. The obtained results were transformed into a binary matrix, and PopGene v. 1.32 (Yeh et al., 2000) was applied to determine such genetic diversity indices as the effective number of alleles per locus; Shannon’s index; expected heterozygosity; Nei’s genetic distance (Nei, Li, 1979). Polymorphism information content (PIC) for every pair of primers was calculated by the formula presented in the study (Chesnokov, Artemyeva, 2015). To build the genetic similarity dendrogram, the unweighted pair group method with arithmetic averages was applied in NTSYSpc v 2.10 (Rohlf, 2000).

## Results

**Fig. 1.**
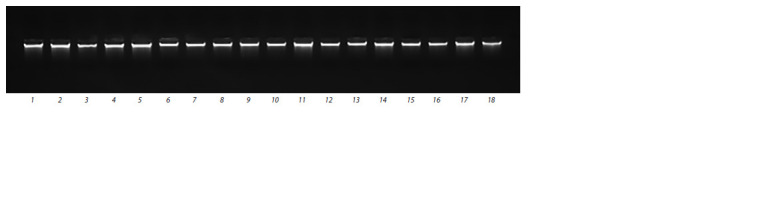
Electrophoregram of the gDNA extracted from the rapeseed and turnip rape germinants. Lanes 1–15 (rape varieties): Severyanin, Stolychniy, VIK 2, Nord, Laureat, Gorizont, Garant, Vikros, Novik, Novosel, Veles, Grant, Podmoskovniy, Lugovskoy, Bizon; 16–18 (turnip rape varieties): Zarya, Nadezhda, Svetlana.

**Fig. 2.**
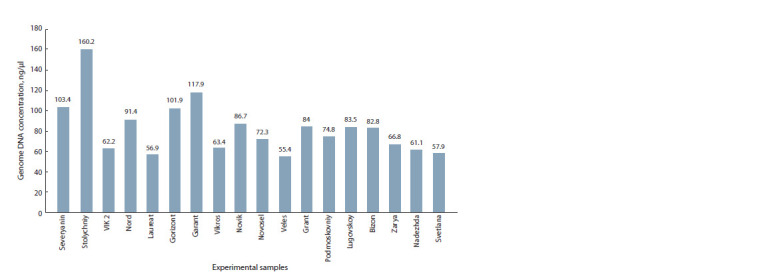
gDNA concentrations.

To obtain gDNA from the rapeseed and turnip rape germinants, a modified SDS method was used. The applied protocol proved more effective and less costly compared to other known protocols and commercial reagents kits. The results of electrophoresis and spectrophotometry attested to the DNA’s high concentration and purification degree from protein compounds and polysaccharides for all experimental samples (Fig. 1, 2).

SSR-analysis

For genotyping the full variety collection, out of 42 SSR primers, 7 primers providing stable and reproducible amplification were selected (Table 1).

**Table 1.**
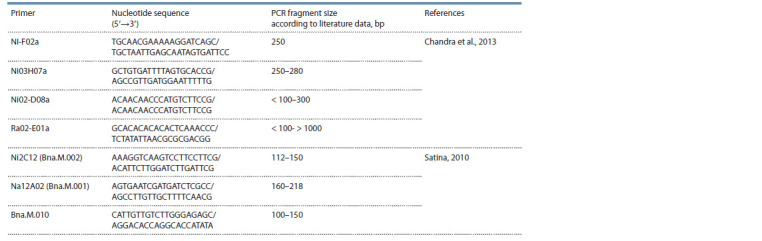
SSR primers used for rapeseed and turnip rape DNA polymorphism analysis

Analysis of the amplification fragments obtained using the listed primers detected 42 alleles. Their number per locus was 6 on average, varying from 3 (Ni2C12 and Bna.M.010) to 10 (Ra02-E01a). The fragment size varied from 110 bps (Ni2C12) to 1200 bps (Ni02-D08a). The maximum allele frequency was registered for Bna.M.010 (0.83), and the minimum – for Ni03H07a (0.27); the mean value was 0.42. The primers developed for Ni03H07a, Ni02-D08a and Ra02-E01a markers made it possible to detect 8–10 alleles per locus and had the highest PIC (0.82).

SRAP-analysis

Based on the results of preliminary testing, the initial 25 combinations of SRAP primers were reduced to 10 pairs, amplifying stable polymorphic DNA fragments (Table 2). In total, 53 PCR fragments of 132–1674 nucleotide pairs in size were obtained. One combination contained from 4 (F9-R9) to 7 (F10-R8, F11-Em2, F10-R7) amplicons. A part of the markers proved to be informative to detect the amplification fragments for differentiating the type of plants (winter/ spring). Using 6 combinations made it possible to obtain the amplicons specific for varieties identification (marked with a star in the Table 2).

**Table 2.**
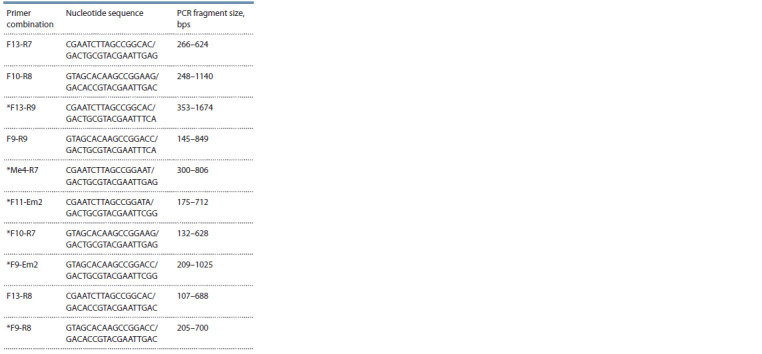
SRAP primers used for rapeseed and turnip rape DNA polymorphism analysis

**Fig. 3.**
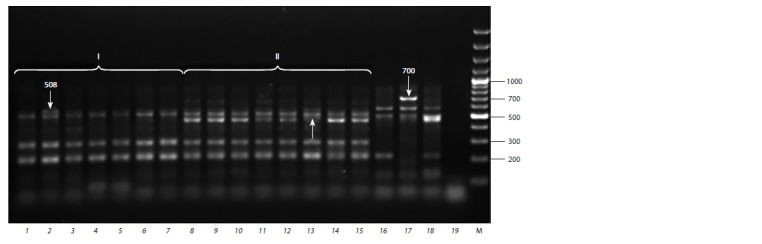
Electrophoregram of the PCR products obtained during amplification of rapeseed and turnip rape varieties with SRAP primers F9-R8. Winter rapeseed varieties: Severyanin (1), Stolychniy (2), VIK 2 (3), Nord (4), Laureat (5), Gorizont (6), Garant (7); spring rapeseed varieties: Vikros (8), Novik (9), Novosel (10), Veles (11), Grant (12), Podmoskovniy (13), Lugovskoy (14), Bizon (15). Winter turnip rape: Zarya (16); spring turnip rape: Nadezhda (17), Svetlana (18). H2O control (19). M – molecular weight marker (100 кb DNA Ladder).

Fig. 3 demonstrates the electrophoregram of PCR results with the F9-R8 primer combination. Significant DNA profile differences were found between winter (I) and spring (II) rapeseed varieties (joined in curly brackets). The arrows mark the variety-specific PCR products characteristic for Stolychniy winter rapeseed (508 bps) and Nadezhda spring turnip rape (700 bps) as well as the absence of an amplicon in size of 460 bps in spring rapeseed Podmoskovniy though it was a specific characteristic for other varieties in this group. The performed analysis demonstrated that it is possible to identify rapeseed varieties Grant and Novosel with 3 marker combinations (F11-Em2, F10-R7 and Me4-R7), and Gorizont and Lugovskoy – with 2 (F13-R9 and Me4-R7). Variety VIK 2 was identified with SRAP primers F9-Em2, and spring ones Veles – with F10-R7. Specific DNA spectra for rapeseed varieties Stolychniy, Podmoskovniy and turnip rape Nadezhda were obtained with F9-R8 combination.

The performed analysis demonstrated that it is possible to identify rapeseed varieties Grant and Novosel with 3 marker combinations (F11-Em2, F10-R7 and Me4-R7), and Gorizont and Lugovskoy – with 2 (F13-R9 and Me4-R7). Variety VIK 2 was identified with SRAP primers F9-Em2, and spring ones Veles – with F10-R7. Specific DNA spectra for rapeseed varieties Stolychniy, Podmoskovniy and turnip rape Nadezhda were obtained with F9-R8 combination

The obtained data were transformed into a binary matrix to calculate Nei’s genetic distances (Table 3). The lowest genetic similarity coefficient (0.7069) was found between rapeseed varieties Gorizont, Novosel and Grant, the highest – between spring varieties Vikros and Bizon (1.0) as well as Veles and Bizon (0.9655). A similarly high genetic distance (0.3228) indicated significant differences between pairs: Grant and VIK 2, and Lugovskoy and Stolychniy. Low distance values and high genetic similarity were demonstrated by spring varieties Bizon and Vikros (zero distance) and winter varieties Garant, Severyanin, Stolychniy, Nord, Laureat (0.0174).

**Table 3.**
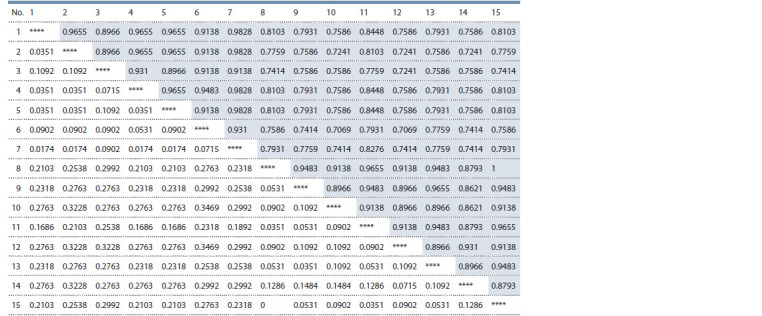
Genetic similarity indices (above the diagonal) and Nei’s distances (below the diagonal) calculated from SRAP analysis results Notе. According to the data of 1 (Rhouma et al., 2017); 2 (Сатина, 2010); 3 (Chandra et al., 2013).

The results of PCR analysis for SSR and SRAP markers were used to determine the genetic variability indices and build an UPGMA dendrogram depicting the varieties’ phylogenetic relationships. The variety material had a low degree of genetic heterogeneity, while higher values of expected heterozygosity (He) and the number of effective alleles (ne) were determined with SSR markers: 0.25 on average against 0.14 and 1.47 per locus if compared to 1.24, respectively. However, the SRAP method has enabled obtaining more PCR products applicable for varietal differentiation (Table 4).

**Table 4.**
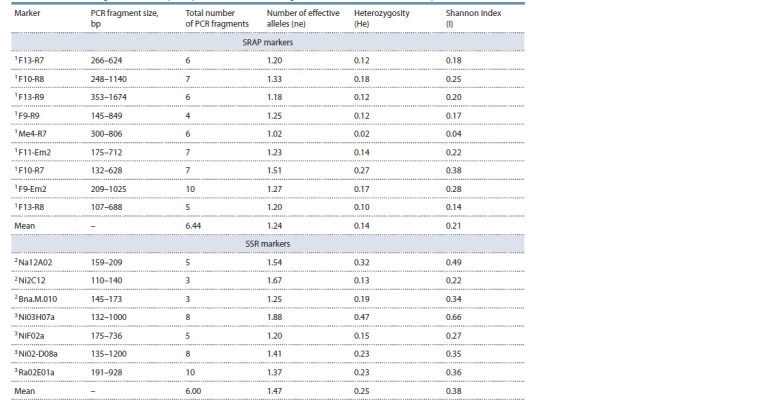
The indexes of genetic variability of rapeseed varieties according to the results of SSR and SRAP analysis Notе. No. 1–15 – rapeseed varieties Severyanin, Stolychniy, VIK 2, Nord, Laureat, Gorizont, Garant, Vikros, Novik, Novosel, Veles, Grant, Podmoskovniy, Lugovskoy, Bizon.

**Fig. 4.**
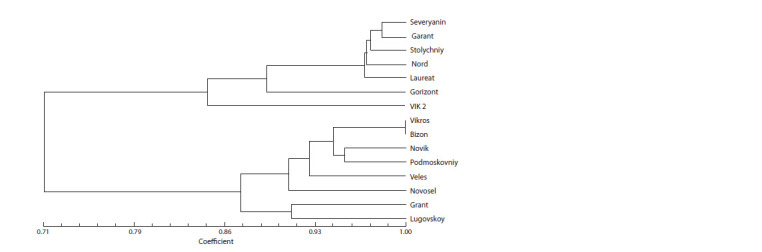
Genetic similarity dendrogram for rapeseed varieties of Federal Williams Research Center of Forage Production and Agroecology.

Analysis of the UPGMA dendrogram demonstrated that the winter/spring rapeseed varieties were divided into two distinguishable clusters (Fig. 4). The first one united such winter cultivars as Severyanin, Garant, Stolychniy, Nord, Laureat, Gorizont, VIK 2; the second – all the spring ones. In the winter cluster VIK 2 and Gorizont were the most distant from the other varieties. The distances between Stolychniy, Nord, Laureat as well as between Garant and Severyanin were much shorter, which was confirmed by their high genetic similarity indices being 0.9655 and 0.9828, respectively (see Table 3). The most distant among spring rapeseed were twozero varieties Novosel, Grant and Lugovskoy, which had the longest genetic distances in the cluster (0.3469 and 0.3228). Bizon and Vikros belonged to one subgroup, sharing a common branch of the dendrogram.

## Discussion

The bulk strategy of DNA sampling from 30 germinants per variety has significantly reduced the labor efforts and cost of the research if compared to the traditional method of individual sample genotyping. The method has proved its efficiency for different cultures especially in large-scale studies of vast populations (Liu et al., 2018). However, this approach is only justified if the analyzed set of samples is representative. For cross-pollinating species with a high level of intrapopulation variations, it should include at least 30–50 plants per variety, which significantly increases the likelihood of registering a rare alleles, the occurrence of which in the population does not exceed 10 % (Crossa, 1989; Semerikov et al., 2002). The plants of winter rapeseed are known for their high self-pollination capacity (up to 70 % of flowers) (Shpaar, 2012), many varieties are linear; while in spring rapeseed this capacity reaches 40 % (Osipova, 1998). That’s why in our study we used budk samples that combined 30 seedlings from each variety.

A significant part of SSR primers tested in our study generated monomorphic amplification fragments. They did not allow us to properly estimate the genetic variability and had low reproducibility in replicated experiments. A proportion of the markers proven effective for intervarietal DNA polymorphism detection comprised 16.7 %, being much lower than in other studies (Plieske, Struss, 2001; Hasan et al., 2008; Tian et al., 2017). It was probably due to the composition of the tested collection that had a narrow genetic basis considering the varieties’ pedigree. At the same time, such parameters of genetic variability as the number of allelic variants, singleallele frequency, PIC and He were comparable to those found in published data (Satina, 2010; Klyachenko et al., 2018).

In general, the used markers made it possible to detect DNA polymorphism between rapeseed and turnip rape as well as between the winter and spring varieties within each species. However, Na12A02 marker turned out to be variety-specific for Bizon winter rapeseed and Zarya spring turnip rape, and Ra02-E01а – for VIK 2 winter rapeseed and Svetlana spring turnip rape. The unique alleles of Podmoskovniy and Lugovskoy rapeseed were detected using Ni02-D08a loci. The indicated markers can be used for varietal DNA identification and genetic certification.

SSR primers for the markers of Indian mustard’s Alternaria blight resistance genes (Chandra et al., 2013), such as Ni02- D08a, Ni03H07a and RA02-E01a, proved to be the most effective. Their application enabled us to detect the specific amplification fragments for linear winter rapeseed variety VIK 2. They also proved effective for Gorizont, which had been obtained on the base of VIK 2 by seed freezing followed by their selection at low-temperature stress. These two varieties share high winter hardiness and are resistant to Alternaria blight. Thereby the results of our study can be useful for further selection of perspective breeding material and QTL analysis on disease resistance.

Among the spring rapeseed, Veles variety turned out to be substantially different while Lugovskoy and Garant had many similarities in the studied microsatellite parts of regions of the genome. Veles is a new perspective variety that has been approved for use since 2021 and was selected based on Vikros using the method of chemical mutagenesis, producing a high frequency of nucleotide changes. This is possibly the reason for Veles having unique alleles in three loci: Ni2C12, Ra02- E01a, Na12A02. For Vikros variety, a specific DNA profile was also obtained with Ni2C12 marker.

Rapeseed Grant was selected using the method of interspecies and intervarietal hybridization of early-maturing foreign breeding samples and the high-yielding varieties Lugovskoy and Vikros, developed at Federal Williams Research Center of Forage Production and Agroecology. Their common origin is probably the reason for the genetic similarity found between Grant and Lugovskoy varieties.

In general, SSR analysis failed to achieve optimum effect in identification of the investigated varieties: from the total set, including 42 primers for microsatellite genome loci, only four were attested as variety-specific for rapeseed, and only one (Ni03H07а) – for Nadezhda spring turnip rape.

For further investigation of DNA polymorphism, SRAP analysis was applied. SRAP is the third generation of molecular markers that were initially designed for the genes of B. oleracea L. (Li, Quiros, 2001) and are successfully used these days for genetic variability estimation and genetic mapping in different plants (Aneja et al., 2012; Rhouma et al., 2017; Liu et al., 2018). This is a cheap, effective and highly reproducible technique

2017; Liu et al., 2018). This is a cheap, effective and highly reproducible technique

The final dendrogram of phylogenetic relations made it possible to visually estimate the degrees of genetic similarities and differences of the studied material. For instance, close placing of such rapeseed varieties as Stolychniy, Nord and Laureat was probably determined by the features of their origin: they were selected for winter hardiness from a combination, in which one of the parental forms was Promin’, a well-known winter rapeseed variety

Garant, selected for winter hardiness, and Severyanin, which was obtained by seed freezing in a climatic chamber and the following individual-family selection, turned out to be in the common subgroup and at a short genetic distance (0.0174) from each other. In addition to high winter hardiness, these varieties are resistant to lodging and to damage by pathogenic fungi

A two-zero spring variety Novosel takes a special position in his group (Nei’s distance is 0.3469). Novosel was developed based on the foreign breeding samples and Russian varieties Lugovskoy and Vikros, characterized by early maturing and high yield. Specific properties of the new breeding achievement are shorter maturation period in comparison to standard varieties and high resistance to Alternaria blight.

Spring rapeseed Bizon and Vikros take the common branch of the dendrogram. The varieties were developed using the method of interspecies hybridization but from different parental forms; characterized by high yield productivity, early maturation and low glucosinolate content.

## Conclusion

The presented study has proved the efficiency of SSR and SRAP markers for estimation of DNA polymorphism in rapeseed and turnip rape varieties developed in Federal Williams Research Center of Forage Production and Agroecology. During the study, SRAP technique has demonstrated a higher level of informativity: 36 % of the tested markers were polymorphic, while for the microsatellite loci this rate did not exceed 16.7 %.

Both techniques of molecular analysis enabled detecting the DNA markers for identification of 10 out of 15 rapeseed varieties tested and for 2 turnip rape samples. Microsatellite loci Na12A02, Ni2C12, Ra02-E01 and Ni02-D08a allowed obtaining unique PCR products for Bizon, Veles, Vikros, VIK 2, Podmoskovniy and Lugovskoy rapeseed varieties. Marker Ni03H07а proved effective for identifying Nadezhda turnip rape. In the used SRAP test kit, such primers as F13-R9, Me4- R7, F11-Em2, F10-R7, F9-Em2 and F9-R8 proved effective for detecting variety-specific amplicons or obtaining unique DNA profiles for different types of plants (winter/spring) in rapeseed varieties Grant, Novosel, Gorizont, Stolychniy, Lugovskoy, Podmoskovniy and in spring turnip rape Svetlana.

The results of the study can be used for development of the perspective breeding samples and hybrids, for genetic certification and seed material purity control.

## Conflict of interest

The authors declare no conflict of interest.
